# Explainable Vision Transformer with Self-Supervised Learning to Predict Alzheimer’s Disease Progression Using 18F-FDG PET

**DOI:** 10.3390/bioengineering10101225

**Published:** 2023-10-20

**Authors:** Uttam Khatri, Goo-Rak Kwon

**Affiliations:** Department of Information and Communication Engineering, Chosun University, 309 Pilmun-daero, Dong-gu, Gwangju 61452, Republic of Korea; uttamkhatri03@gmail.com

**Keywords:** Alzheimer’s disease, vision transformer, ELM, FDG-PET, self-supervised learning, DINO

## Abstract

Alzheimer’s disease (AD) is a progressive neurodegenerative disorder that affects millions of people worldwide. Early and accurate prediction of AD progression is crucial for early intervention and personalized treatment planning. Although AD does not yet have a reliable therapy, several medications help slow down the disease’s progression. However, more study is still needed to develop reliable methods for detecting AD and its phases. In the recent past, biomarkers associated with AD have been identified using neuroimaging methods. To uncover biomarkers, deep learning techniques have quickly emerged as a crucial methodology. A functional molecular imaging technique known as fluorodeoxyglucose positron emission tomography (18F-FDG-PET) has been shown to be effective in assisting researchers in understanding the morphological and neurological alterations to the brain associated with AD. Convolutional neural networks (CNNs) have also long dominated the field of AD progression and have been the subject of substantial research, while more recent approaches like vision transformers (ViT) have not yet been fully investigated. In this paper, we present a self-supervised learning (SSL) method to automatically acquire meaningful AD characteristics using the ViT architecture by pretraining the feature extractor using the self-distillation with no labels (DINO) and extreme learning machine (ELM) as classifier models. In this work, we examined a technique for predicting mild cognitive impairment (MCI) to AD utilizing an SSL model which learns powerful representations from unlabeled 18F-FDG PET images, thus reducing the need for large-labeled datasets. In comparison to several earlier approaches, our strategy showed state-of-the-art classification performance in terms of accuracy (92.31%), specificity (90.21%), and sensitivity (95.50%). Then, to make the suggested model easier to understand, we highlighted the brain regions that significantly influence the prediction of MCI development. Our methods offer a precise and efficient strategy for predicting the transition from MCI to AD. In conclusion, this research presents a novel Explainable SSL-ViT model that can accurately predict AD progress based on 18F-FDG PET scans. SSL, attention, and ELM mechanisms are integrated into the model to make it more predictive and interpretable. Future research will enable the development of viable treatments for neurodegenerative disorders by combining brain areas contributing to projection with observed anatomical traits.

## 1. Introduction

Alzheimer’s disease (AD) is a progressive and irreversible neurodegenerative disorder that primarily affects memory, cognition, and behavior [1]. It is the most common cause of dementia among the elderly, accounting for a substantial global health burden [2]. Currently, there are about 90 million people who have been diagnosed with AD, and it is predicted that by 2050, there will be an estimated 300 million AD patients worldwide [3]. Mild cognitive impairment (MCI), which is a transitional state from normal control (NC) to AD dementia, is frequently regarded as a clinical precursor of AD [4]. Two variants of MCI are often recognized: convertible MCI (MCI-c), which will eventually lead to AD, and stable MCI (MCI-s), which will not. Since there is currently no effective treatment for AD, accurate diagnosis and early detection at the prodromal stage are essential for patient care and the development of future therapies. As a result, patients may begin preventative interventions to delay or stop the disease’s progression if the conversion process from MCI to AD can be accurately predicted. Alzheimer’s is characterized by the accumulation of abnormal protein deposits in the brain, leading to the deterioration and loss of nerve cells [5]. As the world’s population continues to age, understanding and finding effective treatments for AD have become pressing challenges in modern healthcare. For the early detection of diseases in people with mild or no cognitive impairment, AD biomarkers can be used [6,7]. One of the causes of AD, amyloid accumulation in the brain, is known to happen when an abnormal form of amyloid is deposited in the brain as a result of a metabolic issue [8]. An amyloid biomarker is injected into the body as part of an amyloid positron emission tomography (PET) test, which produces a brain image and reveals the location and volume of the deposited amyloid. It serves as an effective functional imaging tool to aid doctors in the diagnosis of AD. As a result, 18F-FDG-PET brain imaging has become one of the potent functional biomarkers for AD diagnosis in clinical and computer-assisted diagnosis (CAD) [9,10,11,12]. In order to identify the patterns associated with AD and decode the disease states for CAD, a number of pattern recognition techniques have been investigated in recent years for analysis of 18F-FDG-PET brain images [13,14,15,16,17].

As researchers and healthcare professionals strive to improve the early detection and management of AD [18,19] the integration of cutting-edge artificial intelligence (AI) [20] techniques has emerged as a promising avenue for advancing diagnostic accuracy and understanding disease progression [21,22,23]. In this journal, we explore the potential of Vision ViTs and 18F-FDG PET in the context of AD research. CNNs have revolutionized various computer vision tasks, demonstrating exceptional performance in image recognition and classification [24,25,26]. These deep learning models have shown promise in medical image analysis [21,27,28], including the interpretation of neuroimaging data, such as magnetic resonance imaging (MRI) and PET scans, which are crucial for diagnosing and monitoring AD [23,29,30,31]. On the other hand, ViT [32,33], a recent breakthrough in deep learning has also gained attention in the computer vision community. These models rely on self-attention mechanisms to learn meaningful hierarchical representations from images, making them effective in handling large-scale image datasets [33]. In the computer vision domain, the self-attention mechanism has shown promising results in tasks such as image classification, object detection, and image captioning. By incorporating self-attention into computer vision models, researchers aim to capture long-range dependencies in images and improve their ability to understand complex visual patterns. This migration has opened new possibilities for advancing computer vision research and pushing the boundaries of what is achievable in visual understanding tasks. Given their strong generalization capability and efficient use of computational resources, ViTs may offer promising results and open new possibilities for improving Alzheimer’s recognition [34]. The AD recognition problem is approached using a supervised method in all the previously mentioned techniques. To overcome these limitations, we have explored SSL techniques for AD recognition. SSL does not require annotated samples and can potentially reduce the cost of data collection. Additionally, simpler model architectures can be used in unsupervised learning, leading to faster training and convergence while reducing the number of parameters that need to be tuned.

This journal investigates the application of ViTs in AD-related tasks, such as early detection, disease progression prediction, and biomarker identification. We discuss their strengths and limitations in handling neuroimaging data and explore how combining the strengths of ViTs may lead to more accurate and interpretable results. Additionally, we delve into the emerging area of explainable AI in AD research, in which the understanding of model decisions becomes paramount for clinical acceptance and integration. The exploration of ViTs in AD research opens new possibilities for improved diagnostics and personalized treatment strategies [35,36,37,38,39]. By harnessing the power of AI, we aim to enhance our understanding of this complex neurodegenerative disorder and pave the way for more effective interventions to improve the quality of life for individuals affected by AD. Traditional methods for predicting the progression from MCI to AD have often relied on supervised learning techniques, for which labeled data are required to train algorithms. However, obtaining sufficient labeled data for such complex neurological conditions can be challenging, time-consuming, and expensive, restricting the development of accurate and generalized predictive models. In recent years, self-supervised learning has emerged as a promising alternative [40,41,42,43] for harnessing the unlabeled data available and enabling the extraction of meaningful representations from medical images without the need for explicit annotations [44,45]. The DINO [43] approach has gained popularity due to its effectiveness in self-supervised learning. It introduces a novel training framework that combines both instance discrimination and clustering objectives. By leveraging these two objectives, DINO achieves state-of-the-art performance on various downstream tasks, such as image classification and object detection. Additionally, the DINO approach also demonstrates strong generalization capabilities across different datasets and domains, making it a promising choice for our research. This discovery suggests that ViTs have a unique ability to capture meaningful visual representations without relying on handcrafted features or explicit supervision. Using the ImageNet image classification dataset, DINO performed exceptionally well and outperformed earlier CNN-based self-supervised methods at a much-reduced computational cost. The ViT model, which has an intriguing feature when compared to CNNs trained in the same manner, serves as the foundation for this method [43]. Self-supervised learning is a type of unsupervised learning in which the algorithm formulates tasks that involve predicting certain aspects of the data using its inherent structure. These tasks effectively generate pseudo-labels or supervise the learning process implicitly, leading to the development of powerful representations that can later be fine-tuned for specific downstream tasks, such as predicting disease progression. By applying self-supervised learning techniques to the study of MCI to AD progression, we have made significant strides in unraveling the underlying patterns and mechanisms that govern this complex transition.

Our method leverages the general ViT architecture as a backbone model to learn valuable Alzheimer’s features from individual 18F-FDG-PET images via the DINO self-supervised learning. These features can then be fed into an ELM classifier to classify individuals. We proved the superiority of the approach in terms of algorithm performance and many medical metrics, including accuracy, specificity, sensitivity, and precision, by validating the suggested framework using the Alzheimer’s Disease Neuroimaging Initiative (ADNI) dataset. Through this approach, the algorithm learns to discover meaningful biomarkers, subtle cognitive changes, and other relevant factors that might contribute to disease progression. The three things that best describe this paper’s contributions are summarized below:

- A transformer model is suggested for the identification of MCI progression. The model expands upon the ViT backbone by utilizing 18F-FDG-PET and self-supervised learning to tackle the issue of MCI progression and disease identification.
- To address the issue of inadequate data in the field of brain imaging, we suggested a cross-domain transfer learning technique. We used ViT as the backbone with DINO.
- In the MCI recognition, experimental data show that the proposed method can achieve more competitive outcomes than current models. The model accuracy levels with the ADNI dataset were 92.31%, which is higher than the baseline’s ViT approach. Finally, we visualized important metabolic brain regions, which can assist the physician for proper analysis of MCI.

## 2. Materials and Methods

**Figure 1:**
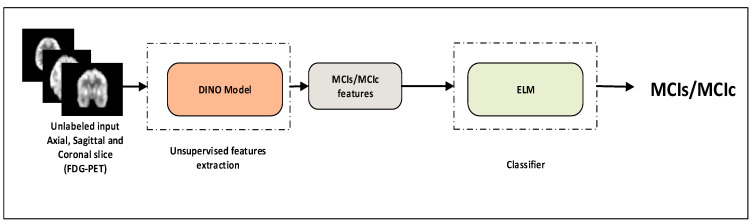
Overall architecture for computer-aided self-supervised Alzheimer’s diagnosis system using ViT-DINO and ELM model.

Figure 1 depicts the three-step method that makes up this study’s framework. Initially, we preprocessed the PET data that had been collected, mostly taking care of partial volume effects (PVE) correction, smoothing, skull-stripping and normalization. In the comparative experiment, we utilized a self-supervised feature extraction method known as DINO with a ViT backbone to learn brain 18F-FDG-PET images. Additionally, we employed t-SNE (t-distributed stochastic neighbor embedding) feature visualization with a different classification algorithm called extreme learning machine (ELM), k-nearest neighbors (KNN), and support vector machine (SVM) to evaluate its effectiveness in classifying MCI-s and MCI-c. The results of experiments are presented and discussed in the results sections.

### 2.1. Dataset

Based on a public–private partnership led by Principal Investigator Michael W. Weiner, MD, the ADNI database (http://adni.loni.usc.edu/ 27 September 2022) provided all the data. ADNI has made significant contributions to our understanding of the early stages of Alzheimer’s disease. The study has provided valuable insights into the biomarkers and cognitive assessments that can help in the early detection and monitoring of MCI and AD. The main objective of ADNI was to determine whether the progression of early AD and mild cogMCI could be monitored using a combination of clinical and neuropsychological assessment, PET, other biological markers, serial magnetic resonance imaging (MRI), and other analyses. This study is multicenter and longitudinal in nature, involving over 63 participating centers. The website http://www.adni-info.org (27 September 2022) offers a comprehensive range of resources, including research findings, study protocols, and data access instructions. Additionally, it serves as a platform for researchers to collaborate and share their findings in the field of AD and related disorders.

We acquired PET scan data from the ADNI 1, ADNI 2, and ADNI GO cohorts in the ADNI database for this study, comprising 224 MCI-c and 245 MCI-s. Following a minimum of 36 months of clinical follow-up, eligible participants with MCI underwent clinical cognitive evaluations and FDG-PET scanning at baseline. Table 1 displays the demographic information of the dataset, which includes age, gender, sex, education, and results from neuropsychological cognitive assessment tests like the dementia rating scale (CDRSB). It also includes information about the apolipoprotein E (APOE) ε4 genotyping characteristics. The groups’ ages did not differ much. The MMSE and CDR did, however, vary across all group pairings (*p* ˂ 0.05). It revealed that compared to MCI-s, MCI-c patients had a higher probability of developing AD. Male dominance prevails in all groups, and the male-to-female ratio is 53:47. Furthermore we also listed the ADNI diagnostic criteria for MCI-s and MCI-c below which can be found details on ADNI website mentioned above.

MCI-s criteria: MMSE scores between 24–30 (inclusive), a subjective memory concern reported by subject, informant, or clinician, objective memory loss measured by education-adjusted scores on delayed recall of one paragraph from Wechsler Memory Scale Logical Memory II (≥16 years: 9–11; 8–15 years: 5–9; 0–7 years: 3–6), a CDR of 0.5, absence of significant levels of impairment in other cognitive domains, essentially preserved activities of daily living, and an absence of dementia.

MCI-c criteria: MMSE scores between 24–30 (inclusive), a subjective memory concern reported by subject, informant, or clinician, objective memory loss measured by education-adjusted scores on delayed recall of one paragraph from Wechsler Memory Scale Logical Memory II (≥16 years: ≤8; 8–15 years: ≤4; 0–7 years: ≤2), a CDR of 0.5, absence of significant levels of impairment in other cognitive domains, essentially preserved activities of daily living, and an absence of dementia.

### 2.2. FDG-PET Image Acquisition and Preprocessing

The ADNI project’s web page contains comprehensive information about the PET acquisition procedure. Thirty minutes after injecting 185 ± 18.5 MBq FDG, 469 cases underwent dynamic 3D scans with six 5 min frames. Each frame was motion-corrected to the first frame and then summed to produce a single image file.

Individual PET scan preprocessing [46] was carried out using MatlabR2021a and the statistical parametric mapping (SPM12) [47] program. Prior to applying PVE correction based on the Muller–Gartner algorithm, PET images were first co-registered with the matching T1-weighted images [48]. This was done to reduce the PVE on PET measurements. The images were then spatially normalized to a PET template in the Montreal Neurological Institute (MNI) brain space using linear and non-linear 3D transformations. The individual anatomical variations were blurred, and the signal-to-noise ratio was increased for further analysis by smoothing the normalized PET images using an 8 mm full-width at half-maximum (FWHM) Gaussian filter over a 3D space. Lastly, the intensity of each PET scan was normalized to the average brain uptake globally. With a voxel size of 2 × 2 × 2 mm^3^, the processed images had a spatial resolution of 91 × 109 × 91. Finally, for the purpose of pre-training the model later, each three-dimensional PET image was divided into two-dimensional images by slicing and tiling it to a size of 224 × 224 pixels.

### 2.3. Self-Supervised Learning

**Figure 2:**
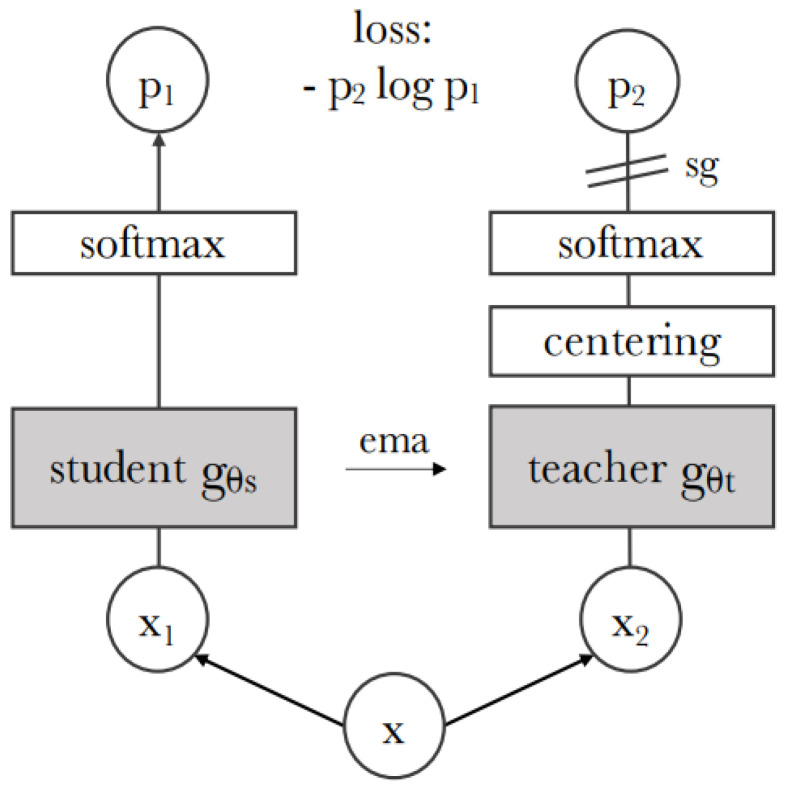
Illustration of self-supervised DINO model [43]. Given many viewpoints of the same input image, the student network’s objective is to use cross-entropy loss to match the probability distribution of a teacher network.

Researchers’ attention has been drawn to self-supervised learning, a new deep learning paradigm, in recent years. The persistent issue of insufficient data for deep learning model training is the focus of self-supervised learning. Through pretext learning—in which one portion of the input data is learned from another portion of the same input—the model learns without labels when it employs self-supervision. Such self-supervised techniques as [40,43] are widely available today. With a contrastive loss function, SimCLR [41] employed contrastive learning by maximizing the similarity between two augmented views of the same image. Two networks—the target network and the online network—with identical architectures but distinct weights were used in BYOL [40]. The target network uses the online network’s exponential moving average to update its weights while the target network trains the latter. Using instance-level discrimination, each image or its transformation is treated as a distinct class in SwAV [49]. The technique uses contrastive loss and image augmentation to learn an embedding such that semantically similar images are clustered closer together in the features space. The label-free knowledge distillation method is applied in DINO [43]. The teacher $g_{\theta s}$ and student $g_{\theta t}$ networks make up the DINO framework. They have the same architecture, but $g_{\theta t}$ and $g_{\theta s}$, the respective parameters, differ. The objective of the student network is to align with the teacher network’s probability distribution. To generate two global views (roughly 50% of the input image) and multiple local views (less than 50% of the input image) for each input image, the method employs a multi-crop strategy [49] during training. Local and global views both flow through the student network, but the global views flow through the teacher network. The similarity between the output vectors from the teacher and student networks is measured using cross-entropy loss. Using stochastic gradient descent, the student parameters $\theta_{s}$ are learned by minimizing the cross-entropy loss, and the teacher parameters $\theta_{t}$ are defined as an exponential moving average of the student parameters. By doing this, the framework can progressively pick up valuable characteristics from the input images, discovering the global to local correspondences between various perspectives on the same image. Additionally, DINO does not need negative samples, which makes training much easier than it would be with many SSL methods [41,42]. Figure 2 below depicts the general architecture of DINO model proposed in [43], which we utilized to predict the AD progression prediction using 18F-FDG-PET in our method.

### 2.4. Vision Transformer (ViT)

Although the standard transformer model was designed for natural language processing, it was given a one-dimensional sequence of word embeddings as input. When the transformer model is used for the computer vision task of image classification, on the other hand, two-dimensional images are used as the input data. It is necessary to divide the input image—which has dimensions of height H, width W, and number of channels C—into smaller two-dimensional patches to structure the data in a way that is similar to how the input is structured in the NLP domain (that is, as a series of individual words) [33]. The outcome is several patches N=HWP2, each with a resolution of (P,P) pixels. The subsequent procedures are carried out prior to supplying the data to the transformer:

Each patch of an image is flattened to create a vector $X_{n}^{P}$ of length $P^{2}\times C$, where, n=1,2,3,…,N. By using a trainable linear projection to map the flattened patches to dimensions D, a series of embedded image patches E is produced. Following this, the series of embedded image patches is appended a learnable class embedding $X_{clas}$. The categorization output y is represented by the value of $X_{class}$. The final step involves adding one-dimensional positional embeddings $E_{pos}$ to the patch embeddings. This adds positional information to the input, which is also learnt during training. Following the previously specified operations, the following array of embedding vectors is produced:

(1) $$Z_{0}=\left[X_{class};\;X_{P}^{1}E;\ldots ;X_{P}^{N}\right]+E_{pos}$$

The sequence of embedding vectors that results from the operations represents the encoded representation of the image patches. This encoded representation captures both spatial and positional information, enabling effective classification and analysis of the image data. Ultimately, a multilayer perceptron (MLP) model receives the transformer encoder’s output class token for categorization. We employ the [43] ViT-B model with patch size 16.

### 2.5. 18F-FDG-PET Feature Learning with ViT-Dino

Our suggested method involves training the feature extractor as the second phase. In this work, we address the challenge of learning discriminative MCI characteristics by proposing to use a self-supervised learning paradigm. We employ the recently suggested DINO approach [43], which has demonstrated promising performance in a range of computer vision applications, including image retrieval and classification. Figure 2 shows the construction of the DINO. Initially, DINO creates two global views of 224 × 224 crops passed via both $\theta_{t}$ and $\theta_{s}$ and eight local views of 96 × 96 crops transmitted exclusively through $\theta_{s}$. Furthermore, since DINO was initially trained on ImageNet, we modified the augmentations applied during training. Specifically, we eliminated most of the image augmentations’ color jitter, Gaussian blur, and solarization and instead used rando horizontal flip, vertical flip, height shift, and random zoom augmentation because the AD related 18F-FDG-PET brain imaging data did not improve performance with the augmentations.

**Figure 3:**
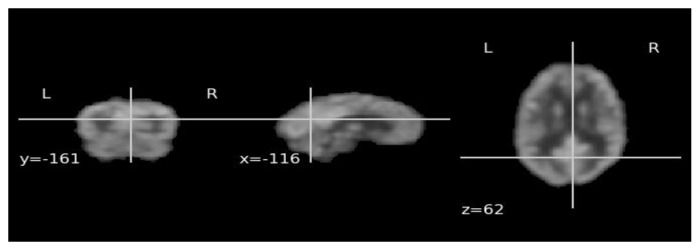
The experiment utilized 18F-FDG-PET ADNI dataset and included illustrations of the coronal, sagittal, and axial slices.

The cross-domain transfer learning technique is employed in this work as AD datasets don’t include the substantial quantity of data required to train the ViT model from scratch [32]. After being trained on the ImageNet dataset, the DINO model is adjusted for Alzheimer’s ADNI data. To generate discriminative features from input brain 18F-FDG-PET for use in classification later, we suggest utilizing the DINO approach as a feature extractor. Figure 3 below illustrates the different slices (coronal, sagittal, and axial) view of input 18F-FDG-PET images to extract the MCI features using ViT DINO architecture for further classification purposes.

### 2.6. Classifiers

**Figure 4:**
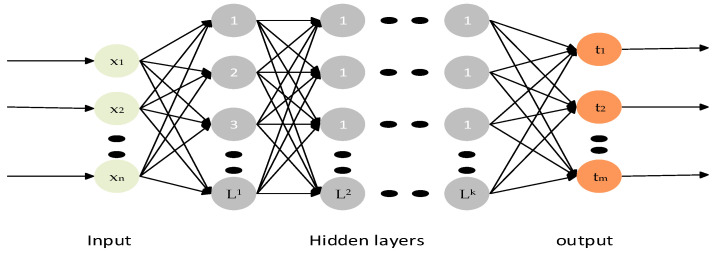
Illustration of multilayer extreme learning machine with multiple hidden layers with input and output layers.

ELM has gained popularity in various fields such as pattern recognition, image processing, and data mining due to its efficient learning process [50]. Additionally, the analytical estimation of output layer parameters eliminates the need for iterative optimization algorithms, making ELM computationally efficient. As a result, gradient-based backpropagation is not needed for the tuning of hidden layer parameters. This makes for incredibly quick training, which makes it especially well-suited for big data analysis. In comparison to traditional neural networks and support vector machines (SVM), ELM has a number of benefits, including quick learning, simple implementation, and little user involvement [51]. Each layer is connected to the layer above it in a feedforward manner, as shown in Figure 4, and creates a feedforward connection with the layer above it.

The multilayer ELM increases the depth of the network by adding extra layers, resulting in improved feature learning capabilities. The multi-layer ELM’s (MLELM) algorithm can be summarized as follows:

ELM algorithm:

For each layer l from 1 to L, randomize the input-to-hidden layer weights.

Calculate the hidden layer output $H_{l}$ for each layer l between 1 and L using the Formula (2):

(2) $$H_{l}=g_{l}(X\times W_{l})$$

where X stands for the input data, $g_{l}$ is the layer l activation function, and $W_{l}$ represent the layer l weight matrix.

To get the final hidden layer output H, combine the outputs of every hidden layer. The following equation represent the output weights:

(3) β=pinv(H)×Y

where pinv(H) represents the Moore-Penrose pseudoinverse of the H output from the hidden layers.

The MLELM algorithm offers a proficient approach to train deep architectures, capitalizing on ELM’s rapid learning capabilities while harnessing the expressive potential of multiple hidden layers. As a result, MLELM adeptly captures intricate patterns and extracts high-level features from intricate datasets, thereby bolstering its classification performance.

### 2.7. Training Setup

Using the official GitHub repository [52], the DINO method was implemented. To optimize the student and teacher networks, the ImageNet pretrained DINO model checkpoint was employed. Only ViT-B models with patch size 8,16, and 32 architecture were employed in our studies. The remaining DINO model parameters are the same as in the original publication [43], including global and local crop scales, teacher temperature, and momentum teacher value.

Using the ADNI 18F-FDG-PET imaging datasets for all experiments, we trained the DINO models for 300 epochs with a batch size of 32. With a learning rate of 0.0001, AdamW [53] was the optimizer that was employed. Python 3.9.13 with a computer equipped with an Nvidia GeForce RTX 3090 GPU and the Windows 10 × 64 operating system was used for the training. The performance of the MLELM classifier is strongly influenced by the number of hidden layer nodes used. In this experiment, we generated extremely accurate performance results using 300 hidden layers. Moreover, we performed 5-fold cross-validation for the robustness of classifier in our models. Since training the model for a longer period did not increase accuracy, the number of epochs used to train the DINO model was fixed at 300 epochs. Using steps to the power of 2, the ideal value for the batch sizes of 32 was found to determine the batch size of models.

### 2.8. Evaluation Matrixs

The findings were assessed using specificity, sensitivity, precision, recall, F1 score, and accuracy; we reported our results in term of mean and standard deviation. These parameters were expressed mathematically as follows:

(4) $$Accuracy=\frac{T_{n}+T_{P}}{T_{n}+T_{p}+F_{n}+F_{p}}$$

(5) $$Sensitivity=\frac{T_{p}}{T_{p}+F_{n}}$$

(6) $$Specificity=\frac{T_{n}}{T_{n}+F_{p}}$$

(7) $$Precission=\frac{T_{p}}{T_{p}+F_{p}}$$

(8) $$Recall=\frac{T_{p}}{T_{p}+F_{n}}$$

(9) $$F1-score=2\times \frac{Precision\times recall}{Precission+recall}$$

True negatives, true positives, false negatives, and false positives are represented by the letters $T_{n}$, $T_{p}$, $F_{n}$, and $F_{p}$, respectively. Concurrently, a receiver operating characteristic (ROC) curve was generated to provide an understandable comparison of the outcomes of the various methodologies.

## 3. Results

This study develops and implements a CAD system which is automated for the diagnosis of AD. The suggested approach was used to distinguish between MCI-s and MCI-c patients progressing to AD. The simulation made use of the 18F-FDG-PET image, which was taken from the ADNI database; 469 patients had their 18F-FDG-PET scans taken, comprising 245 MCI-s patients without conversion within 3 years and 224 MCI-c patients who converted to AD within 3 years.

### 3.1. Classification Performance on 18F-FDG-PET

**Figure 5:**
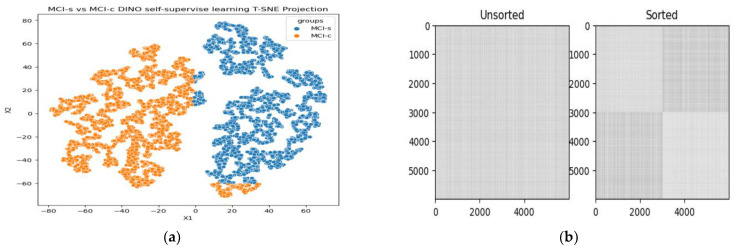
Illustrative visualizations of ViT-DINO model learning: (**a**) t-SNE projections for MCI-s/MCI-c group identification; (**b**) distance matrix between two groups; white means smaller Euclidean distances; and the squares near the white diagonal represent t-SNE and try to roughly preserve the distances between samples.

It is crucial to identify AD in a timely manner for patient care. To distinguish MCI-s from MCI-c, a 2D ViT base DINO model is utilized in this research. The proposed CAD system’s structure is shown in Figure 1. According to Figure 3, each 3D 18F-FDG-PET image is split into several 2D images along the coronal, axial and sagittal axis. The first and last 15 slices are eliminated to remove the skull and other undesirable regions. Table 2, Figure 4 and Figure 5 display how well 18F-FDG-PET-based ViT performed in predicting the transition of MCI to AD.

We utilize transfer learning for ViT by initializing the model with weights that were pre-trained on ImageNet [54] to enhance the model’s performance. However, since the images in ImageNet differ from brain images, many of the weights may not be relevant. To address this issue, we employ a self-supervised pre-training target dataset, which has gained popularity recently due to the lack of a large brain imaging dataset. Our approach incorporates the DINO self-supervised method, which shares a similar overall structure with other self-supervised algorithms. The input images are transformed to generate alternative views, which are then passed through the student and teacher branches. Subsequently, the resulting features are used to compute a loss. The student and teacher networks in DINO have identical structures and initial weight parameters, but the teacher network’s weights are not involved in training and do not have gradients. The parameter updates are based on the student network’s parameters. Additionally, the teacher network includes a phoebe module.

**Figure 6:**
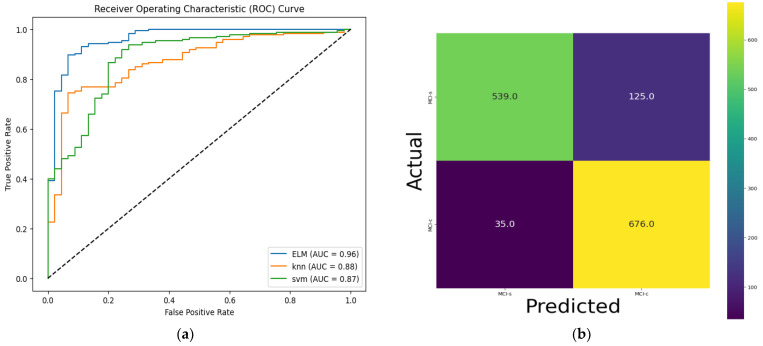

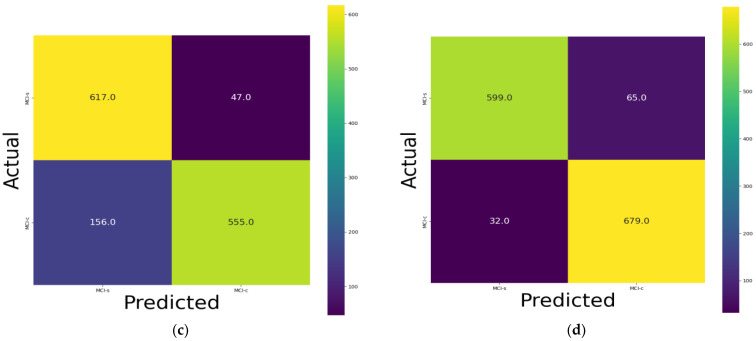
Illustrative visualizations of classification performance of proposed model: (**a**) comparison of ROC curve for different classifiers, (**b**) confusion matrix for KNN classifier, (**c**) confusion matrix for SVM classifier, and (**d**) confusion matrix for ELM classifier.

Furthermore, we utilized the t-SNE algorithm to reduce the complexity of the features obtained from the DINO network and projected them onto a two-dimensional space for visualization purposes. As depicted in Figure 5a, in the MCI conversion prediction, clear boundaries were observed between the two categories. Additionally, only a small number of samples from other categories were scattered within each category, suggesting that the model is more effective at identifying MCI cases without labeling 18F-FDG-PET imaging data. Similarly, as shown in Figure 5b, the model successfully separated the samples into two clusters using Euclidean distance for MCI-s and MCI-c classification. Although a few cases were mixed at the intersection of the two clusters, indicating a transitional stage from MCI-s to MCI-c, our model was able to extract the features between the two stages accurately. We can say DINO successfully applies ViT to self-supervised learning and achieves superior performance compared to baseline ViT for AD dataset. To determine the appropriate model for classifying MCI-c vs. MCI-s, the performance of several models, including the baseline ViT variant and the self-supervised ViT model [43], were compared. The classification results for these models, including accuracy, sensitivity, and specificity, are summarized in Table 2. Since MCI serves as a transitional stage between AD and NC, there are numerous factors that complicate the classification task. It is evident that classifying MCI-s vs. MCI-c is more challenging compared to the other AD classification tasks mentioned earlier [34]. First, we extracted the glucose metabolic features from 18F-FDG-PET images using the ViT-DINO model without labeling data. Secondly extracted features are fed into the different classifiers, namely ELM, SVM and KNN. Specifically, the ELM model achieved an accuracy of 92.31%, a sensitivity of 90.21%, a specificity of 95.50%, an AUC of 0.96, and a 93.92% F1-score. Although KNN achieved comparable specificity of 95.08%, their results were lower in terms of accuracy and sensitivity. Among these models, ViT-DINO with ELM was found to be the most suitable, as it not only had the best classification performance in the independent test group but also had a shorter training time. Therefore, ELM was chosen as the classification model for extracted features in this research. Furthermore, we also evaluated the ROC curve, which is a mathematical tool that evaluates how well a classification system can distinguish between positive and negative cases. It compares the true positive rate to the false positive rate on a ROC chart, which is determined by adjusting the threshold value. Figure 6 displays the ROC of the suggested system, with an AUC value of 0.96. The comparison of ROC curves for different classifiers in the classification of MCI-c and MCI-s can be seen in Figure 6a.

Based on the experimental results, we can say that in our task of early prediction classification for MCI, the DINO model can be used instead of the baseline ViT. We observed that when we used the weights obtained from the DINO self-supervised pre-training on the ADNI dataset, which was initialized by ImageNet, all evaluation metrics of the model improved. The accuracy increased by 5.99%, and the F1 score increased by 4.95% compared to the best-performing baseline ViT model. This suggests that self-supervised learning pre-training enables the ViT model to perform better on a small dataset like ADNI. Furthermore, by incorporating the ELM, the model’s performance improved even more. Each of the four metrics showed varying degrees of improvement, with accuracy reaching 92.31% (a 3.95% improvement) F1 score reaching 93.92% (a 5.67% improvement) as compared to KNN classifiers. This indicates that the ELM classifiers effectively classified the MCI pathology using features extracted from DINO model that was previously overlooked.

### 3.2. Ablation Study

We performed an ablation experiment to examine the impact of transformer design decisions on MCI-to-AD categorization. We investigated the effects of several patch sizes in the experiment. Three variations of patches size were tested: the patch size of 8, the patch size of 16, and the patch size of 32. All models were trained using pre-trained weights from DINO that were included in the Python image model implementation [54]. There were three options for patch size: 8, 16, and 32. Table 3 presents the outcomes of different patch sizes on MCI progression prediction. We noted that the greatest classification performance is provided by the DINO ViT-B, which has a patch size of 16 with 12 attention heads. According to our analysis, the patch size of 16 may capture the 18F-FDG-PET images’ most useful and instructive glucose metabolic aspects. By separating the brain areas with similar patch sizes, the proposed model generates predictions. The information gathered by the model becomes overly generic and loses many specifics with a greater patch size, which results in underfitting. On the other hand, an image patch size that is too tiny might obliterate the 18F-FDG-PET scan’s glucose metabolic information. Detailed studies of our investigation are presented in Table 3 below.

### 3.3. Performance Comparison with State-of-Art Methods

In recent times, there has been significant research conducted on the use of machine learning techniques for predicting MCI stage using brain imaging. Most of these studies have focused on using structural imaging of the brain, with only a few utilizing functional imaging, specifically 18F-FDG-PET. In this section, we are comparing our results with recent findings in the literature from the ADNI database for diagnosing MCI. Some researchers have attempted to analyze 18F-FDG-PET for AD prediction, but these studies have still relied on manual and supervised features extraction [55,56]. Table 4 provides an overview of the latest deep learning methods for predicting AD using neuroimaging techniques. Most of the methods examined can only distinguish between AD and normal control (CN) or mild cognitive impairment (MCI) and CN, whereas our method analyzed the predictive diagnosis of MCI stage. Furthermore, our experiments utilized self-supervised learning compared to these methods, demonstrating the superior generalization capability of our approach.

**Figure 7:**
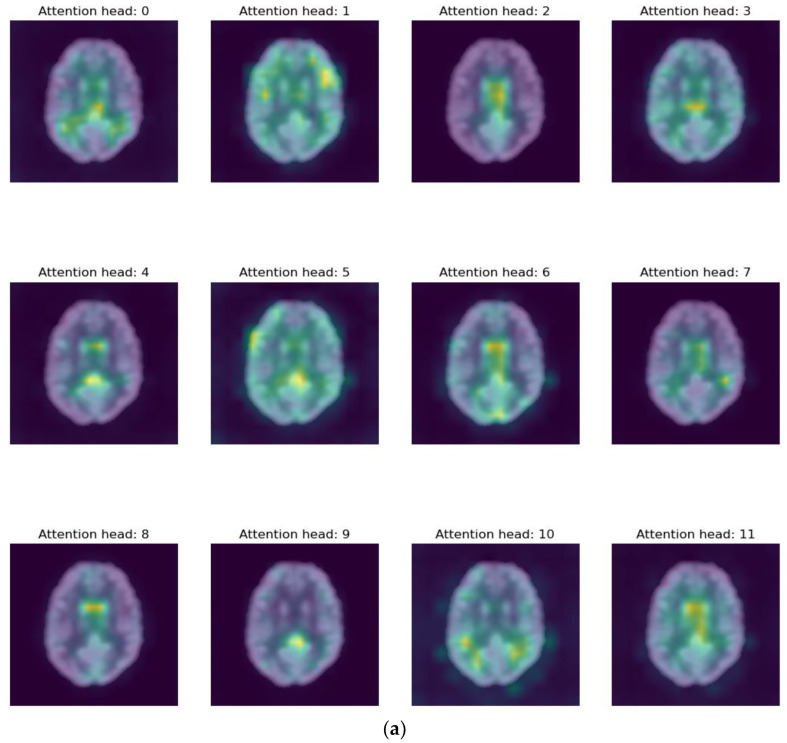

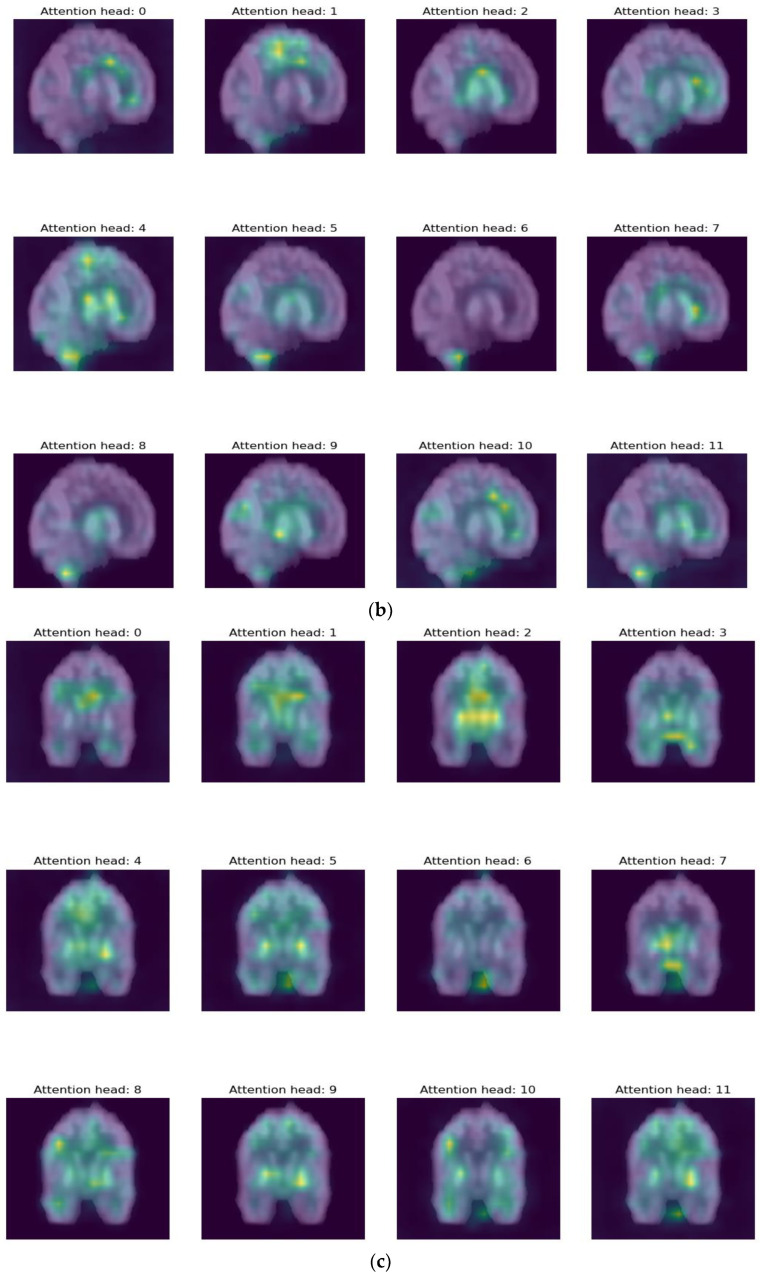
Illustrative visualizations of ViT-DINO attention maps on 18F-FDG-PET glucose metabolic regions, where highlighted regions represented highly sensitive brain area corresponding to each attention head: (**a**) axial slice view, (**b**) sagittal slice view, and (**c**) coronal slice view.

Specifically, we compare our results with five methods described by Nozadi et al. [55], Bae et al. [56], Hoang et al. [34], Duan J et al. [57], and Choi and Jin et al. [58] since they utilized FDG-PET images in their experiments as summarized in Table 4. Nozadi et al. [55] proposed a traditional machine learning method which compared multiple simple classifiers and performed feature selection simultaneously with FDG-PET parcellation to improve classification performance. Bae et al. [56] proposed a CNN with ResNet backbone deep learning, generated in the 3D- space of each subject, to extract regional glucose metabolic area. Hoang et al. [34] extracted mild sagittal-slice-based features of sMRI neuroimages using ViT models for stage of MCI classification. Choi and Jin et al. [58] proposed deep learning achieved an accuracy rate of 84.2% with AUC of 0.89. These methods involve supervised features and voxel-wise feature extraction and traditional classification on FDG-PET and sMRI images from the ADNI database. However, Hoang et al. utilized the latest ViT-based deep learning models in supervised manner. Therefore, most of the state-of-art methods rely on the supervised learning methods for MCI diagnostic classification. To address this issue, we implemented fully automated self-supervised learning in deep learning to identify the MCI stage, which is crucial for timely AD identifications without human intervention. Table 2 and Table 3 and Figure 7 display the results of our FDG-PET-based vision transformers to predict MCI-to-AD progression in a fully automated manner. Table 4 presents our findings as well as those from other studies, including the methodology used and the performance measures. We introduced a self-supervised version of vision transformers along with ELM. Our method consistently outperforms previous studies in three classification performance indicators: sensitivity, specificity, and accuracy. ELM achieves accuracies of 92.31% and 6.05% improvement in comparison to highest-performing Bae et al. [56] study in terms of accuracy. KNN also demonstrates a significant enhancement in specificity with 95.08%. Although their results achieved similar accuracy, their results are lower in specificity and sensitivity. Figure 6 illustrates the confusion matrix of our model, which yields the best result among our methods, with an AUC of 0.96. Ultimately, our proposed method is highly efficient compared to the latest neuroimaging-based research for the predictive diagnosis of MCI.

### 3.4. Pathological Attention Regions on FDG-PET by ViT DINO

For computer-aided diagnosis, identifying the brain area most closely associated with the deep learning model prediction is crucial. Observing the structural change in the brain is one of the most important factors in the clinical diagnosis of AD and the progression of MCI to AD. We study the potential diseased brain area associated with the prediction of our method as a predictive brain region. To categorize MCI-c and MCI-s classes, we employ self-attention visualization [43] to look at which brain regions attention layers see and focus on (Figure 7), which demonstrates glucose metabolic regions in axial, coronal, and sagittal slices that were found using our suggested strategy. The highlighted regions display the corresponding glucose metabolic activities of FDG-PET. Our findings reveal that the thalamus, medial frontal, hippocampus, posterior temporal lobe, parietal lobe, posterior cingulate gyrus, left Para hippocampal gyrus, and occipital regions are the most informative for our model’s prediction. These marked regions align with previous studies on AD diagnosis [34,59,60,61], which supports the reliability of our proposed model.

## 4. Discussion

As the population ages, the number of patients with Alzheimer’s disease continues to rise. However, progress in finding a cure for AD has been slow, leading researchers to focus on early diagnosis to delay the progression of the disease through preventive measures. Nevertheless, identifying patients in the prodromal stage of AD remains a difficult task. A neural-network-based model has shown promise in accurately identifying patients with AD at different stages, surpassing the performance of professional radiologists in terms of sensitivity and specificity. Previous studies have identified a specific pattern of reduced brain metabolism in 18F-FDG-PET scans of AD patients, particularly in the bilateral temporo-parietal regions. As the disease advances, reduced FDG uptake is also observed in the frontal, parietal, and lateral temporal lobes. However, 18F-FDG-PET alone is not a definitive biomarker for AD and MCI. While previous attempts to develop CAD diagnostic methods for AD using other imaging modalities have been made, few studies have focused on using machine learning approaches to classify AD patients based on 18F-FDG-PET scans alone. In addition to predicting AD, our model can accurately classify patients with MCI-s and MCI-c, achieving high sensitivity and specificity. The advantages of our model include its ability to dynamically update without retraining from scratch when new imaging studies are added as well as its superior performance in identifying the early stage of AD. Effective and accurate prediction of MCI transitioning into AD holds utmost importance in facilitating timely intervention and disease management. Consequently, numerous studies undertake endeavors to investigate and enhance the predictive capabilities for MCI progression. In this investigation, a comprehensive comparative analysis was conducted to assess the predictive capabilities of DINO-ELM in utilizing 18F-FDG-PET data from the ADNI. Notably, our proposed method exhibited superior performance when compared to the prevailing state-of-the-art MRI-based studies pertaining to MCI progression diagnosis. With an accuracy rate of 92.31%, specificity rate of 95.50%, and sensitivity rate of 90.21% along with 0.96 AUC, our findings demonstrate the potential of employing vision transformers equipped with attention mechanisms with SSL without any human intervention to achieve heightened classification accuracy in contrast to prevailing CNN architectures. This improvement may be attributed to the attention mechanism within vision transformers effectively highlighting distinctions within the brain regions between MCI-c and MCI-s classes.

Additionally, we have also examined the brain regions that impact the prediction of our proposed method. Discovering these regions will facilitate the future advancement of deep learning models, enhancing their classification performance. Furthermore, it will aid doctors in effortlessly identifying the regions of interest for diagnosis. We have identified primary regions with the highest attention score: the thalamus, medial frontal, hippocampus, posterior temporal lobe, parietal lobe, posterior cingulate gyrus, left Para hippocampal gyrus, and occipital. Notably, 18F-FDG-PET scans have revealed brain atrophy in these regions. Figure 7 illustrates examples of 18F-FDG-PET scans for MCI-c cases. The thalamus serves as the primary relay for sensorimotor information in the brain and is believed to be vital for memory processing, early affected by AD [60]. The medial frontal area also plays a crucial role in various cognitive functions, including attention, spatial perception, and long-term memory [61]. The occipital region, responsible for visual perception encompassing color, form, and motion, experiences volume reduction due to AD [62]. The posterior cingulate gyrus and left parahippocampal gyrus also exhibit consistent involvement [59,63]. These findings imply informative regions for future feature extraction to enhance our proposed method by allocating more attention to these locations. Additionally, these marked brain regions, crucial for the method’s prediction, offer valuable insights for doctors in clinical diagnosis.

Our model has some limitations. Firstly, the training process is complex and needs to be completed in two stages. Additionally, our current method does not utilize a full 3D scan model, instead, it only extracts slices from the brain. This approach may result in missing global anatomical information from other brain regions, which could affect the accuracy of our predictions. The quality of feature extraction in the attention layer also affects the performance of the self-supervised model in the second stage. However, in real clinical scenarios, the causes of hypometabolism observed in 18F-FDG-PET may be more complex. Other types of dementia, such as dementia with Lewy bodies (DLB) or frontotemporal dementia (FTD), can also lead to similar pathological changes such as AD. Further studies on more complex data can provide more reliable clinical aids for the diagnosis of AD. In this work, our study only focuses on MCI to AD progression. In future studies, we should focus on other diagnostic groups, including healthy control, MCI, and AD. Therefore, future studies will focus on incorporating multimodal brain data, including functional MRI (fMRI), structural magnetic resonance imaging (sMRI), and other modalities to identify different diagnostic groups. By integrating multiple imaging modalities, researchers aim to enhance the discriminative power of the models and achieve even better performance in the classification of brain-related conditions.

## 5. Conclusions

In summary, utilizing brain 18F-FDG-PET, our study has created a ViT-DINO-based features extractor network along with an ELM classifier for diagnostic prediction of MCI. Features were extracted by decomposing the 18F-FDG-PET images into 2D slices. The slices were then arranged at a few intervals without overlapping. The ADNI dataset verified the suggested CAD system. This integrated approach demonstrates strong performance in the MCI classification task following pre-training through DINO self-supervised learning. Additionally, results of the simulations clearly showed that the utilization of ELM enables the vision transformer to achieve enhanced performance in AD tasks with superior classification accuracy and resilience. Furthermore, our approach primarily had a profound effect on specific brain regions that were visually portrayed. The thalamus, medial frontal, hippocampus, and occipital regions of 18F-FDG-PET emerged as the pivotal components within our proposed framework. These discoveries highlight the potential for early identification and classification of individuals with MCI, utilizing patterns of functional atrophy as reliable indicators, prior to subjecting them to interventional clinical studies. Future research will concentrate on expanding the recommended CAD system to include data from additional sources to increase the classification accuracy. Several different samples will be used to assess the overall performance of the proposed CAD system.

## Figures and Tables

**Table 1:** Demographic and statistical information regarding clinical assessments at the time data was collected can be found below.

Groups	Gender (M/F)	Education	Age (Years)	MoCA	MMSE	CDR	APOEƐ4
MCI-s	130/115 ^#^	16.3 ± 2.7	72.3 ± 7.5 *	23.7 ± 2.4 *	28.0 ± 1.7 *	1.1 ± 0.5 *	43.1%
MCI-c	119/105	16.2 ± 2.1	74.1 ± 7.3	21.1 ± 2.7	26.3 ± 2.1	2.3 ± 1.0	74.0%

All data except APOEƐ4 positive rate were presented as mean ± standard deviation; education; MMSE = mini-mental state examination; MoCA = Montreal cognitive assessment; CDR = clinical dementia rating. # Group-level two-sample *t*-tests are conducted for age, education, MMSE, MoCA, and CDR; * group-level chi-square tests are conducted for gender.

**Table 2:** Comparison of the proposed model with ViT based studies for predicting the progression of mild cognitive impairment (MCI).

Model	Classifiers	ACC % (mean ± std)	SEN % (mean ± std)	SPE % (mean ± std)	PRE % (mean ± std)	Recall % (mean ± std)	F1-Score % (mean ± std)
ViT-S		82.37 ± 1.29	75.51 ± 2.01	88.71 ± 1.03	83.87 ± 1.45	84.33 ± 3.02	83.30 ± 1.05
ViT-B		81.75 ± 2.13	85.38 ± 3.14	79.85 ± 1.71	83.54 ± 2.52	82.47 ± 2.45	82.71 ± 1.71
ViT-L		78.93 ± 1.07	67.83 ± 2.73	90.97 ± 1.07	81.75 ± 2.59	78.83 ± 3.74	79.34 ± 2.15
DINO ViT-B	KNN	88.36 ± 1.91	81.71 ± 2.47	95.08 ± 1.32	89.15 ± 3.11	88.31 ± 2.14	88.25 ± 1.72
	SVM	85.24 ± 3.73	92.92 ± 1.01	78.06 ± 3.45	86.01 ± 2.47	85.49 ± 1.75	85.21 ± 1.04
	ELM	92.31 ± 1.07	90.21 ± 3.37	95.50 ± 2.15	93.10 ± 1.88	92.95 ± 2.31	93.92 ± 1.33

ACC: accuracy; SEN: sensitivity; SPE: specificity; PRE: precision; std: standard deviation.

**Table 3:** Investigation of the efficiency of different patch sizes of DINO ViT-B for predicting the progression of mild cognitive impairment (MCI).

Model	Patch Size	Classifiers	ACC % (mean ± std)	SEN % (mean ± std)	SPE % (mean ± std)	PRE % (mean ± std)	Recall % (mean ± std)	F1-Score % (mean ± std)
DINO ViT-B	8	KNN	87.49 ± 1.23	95.93 ± 3.11	79.61 ± 2.15	88.45 ± 1.33	87.77 ± 1.04	87.46 ± 1.87
		SVM	86.47 ± 1.55	78.16 ± 2.45	94.23 ± 1.07	87.44 ± 1.58	86.2 ± 2.78	86.31 ± 1.95
		ELM	91.56 ± 1.03	86.75 ± 1.47	96.06 ± 1.56	91.98 ± 1.19	91.4 ± 1.74	91.51 ± 1.51
	16	KNN	88.36 ± 1.91	81.71 ± 2.47	95.08 ± 1.32	89.15 ± 3.11	88.31 ± 2.14	88.25 ± 1.72
		SVM	85.24 ± 3.73	92.92 ± 1.01	78.06 ± 3.45	86.01 ± 2.47	85.49 ± 1.75	85.21 ± 1.04
		ELM	92.95 ± 1.07	90.21 ± 3.37	95.50 ± 2.15	93.10 ± 1.88	92.95 ± 2.31	93.92 ± 1.33
	32	KNN	82.62 ± 3.01	93.52 ± 1.41	72.43 ± 4.75	84.15 ± 2.13	82.98 ± 1.71	82.58 ± 1.37
		SVM	81.31 ± 2.45	64.16 ± 5.78	97.33 ± 1.21	85.07 ± 2.04	80.74 ± 3.41	80.58 ± 1.78
		ELM	86.84 ± 1.58	75.45 ± 3.71	97.47 ± 1.03	88.74 ± 1.23	86.64 ± 1.59	86.57 ± 1.79

ACC: accuracy; SEN: sensitivity; SPE: specificity; PRE: precision; std: standard deviation.

**Table 4:** Comparison of the proposed model with ADNI data-based studies for predicting the progression of mild cognitive impairment (MCI).

Study	Modality	Method	ACC	SEN	SPE
Nozadi et al. [55]	FDG-PET	RF	72.5	79.2	69.9
Bae et al. [56]	MRI	ResNet	86.1	84	74.8
Zhu et al. [28]	MRI	Dual attention multi-instance deep learning network	80.2	77.1	82.6
	MRI	ViT-S	83.27	85.07	81.48
Hoang et al. [34]		ViT-B	80.67	79.1	82.22
		ViT-L	72.86	74.63	71.11
Duan J et al. [57]	FDG-PET	CNN	-	81.63	85.19
Choi and Jin et al. [58]	FDG-PET	Deep Learning	84.2	81.0	87.0
Our	FDG-PET	DINO-ELM	92.95	90.21	95.50

ACC: accuracy; SEN: sensitivity; SPE: specificity.

## Data Availability

The dataset used in this article was obtained from the ADNI webpage, which can be easily accessed from the ADNI websites and is freely available for use by all researchers and scientists conducting research on Alzheimer’s disease: http://adni.loni.usc.edu/about/contact-us/, accessed on 27 September 2022.
